# Choroidal involvement in non-infectious posterior scleritis

**DOI:** 10.1186/s12348-021-00269-9

**Published:** 2021-10-27

**Authors:** Sandra Vermeirsch, Ilaria Testi, Carlos Pavesio

**Affiliations:** Moorfields Eye Hospital, National Health Service Foundation Trust, 162 City Rd, Old Street, London, EC1V 2PD UK

**Keywords:** Posterior scleritis, Choroid, Choroidal involvement, Choroidal thickness, Choroidal vasculitis, Choroidal mass, Choroidal folds, Exudative retinal detachment

## Abstract

**Purpose:**

To provide a comprehensive overview of choroidal involvement in non-infectious posterior scleritis; including different imaging modalities and their clinical usefulness.

**Methods:**

Narrative review.

**Results:**

Posterior scleritis is an uncommon yet potentially sight-threatening inflammation of the sclera. During the disease process, inflammation can spread to the adjacent choroid, causing different manifestations of choroidal involvement: (1) increased choroidal thickness, (2) choroidal vasculitis, (3) presentation as a choroidal or subretinal mass in nodular posterior scleritis, and (4) choroidal folds, choroidal effusion and exudative retinal detachment.

**Conclusions:**

Clinical characteristics and multimodal imaging can aid in diagnosing and monitoring disease progression and response to treatment in non-infectious posterior scleritis with choroidal involvement.

## Introduction

Posterior scleritis is a potentially sight-threatening, inflammatory process of the sclera that primarily involves the posterior segment of the eye [1]. It is an uncommon condition, affecting between 2.0 and 17.7% of all scleritis patients in retrospective epidemiological studies [2–12]. Posterior scleritis is reported to have a female preponderance in the majority of case series and can occur in all decades of life (Table 1) [13–28].

**Table 1:** Literature review of case series on posterior scleritis

Authors (year of publication)	Country	Number of patients	Age at presentation (years)	M:F ratio
Agrawal et al. (2016) [13] ^a^	UK	11	Mean 57 (range 30–84)	1:4.5
Ando et al. (2020) [14]	Japan	10 (13 eyes)	Mean 50.1 (range 17–77)	1:1
Benson W. (1988) [15]	US	43	Median 38 (range 8–75)	1:2.1
Benson et al. (1979) [16]	US	7	Median 43 (range 30–55)	(only F)
Biswas et al. (1998) [17]	India	8	Median 35.5 (range 11–46)	1:1
Calthorpe et al. (1988) [18]	UK	47	Mean 49 (range 12–87)	1:1.5
Cheung et al. (2012) [19] ^b^	Singapore	13 (20 eyes)	Median 11.5 (range 5–16)	1:1.6
Dong et al. (2019) [20] ^c^	China	23	Mean 29.5 (range 19–57)	1:1.3
Gonzalez-Gonzalez et al. (2014) [21]	Spain and US	31	Mean 43.6 (range 12–77)	1:5.2
Gonzalez-Lopez et al. (2016) [22] ^d^	UK and India	18	Median 48 (range 17–83)	1:3.5
Kumar et al. (2018) [23]	India	18	Mean 41.2 (range 26–63)	1:1.2
Lavric et al. (2016) [24]	UK and India	114	Mean 45.9	1:2.5
McCluskey et al. (1999) [25]	UK	137	Mean 49.3 (range 11–84)	1:1.8
Rosenbaum et al. (1993) [26]	US	6	Mean 50.3 (range 33–64)	1:0.2
Singh et al. (1986) [27]	Germany and US	9 (12 eyes)	Mean 40.2 (range 14–68)	1:0.2
Wald et al. (1992) [28] ^b^	US	4	Mean 11.8 (range 10–14)	(only M)

^a^ Nodular posterior scleritis; ^b^ Only children included; ^c^ Posterior scleritis presenting with serous retinal detachment; ^d^ Bilateral posterior scleritis

Diffuse and nodular forms of posterior scleritis can be discerned [25, 29]. The majority of patients with posterior scleritis present with unilateral involvement without associated active anterior scleritis, although more than half of all patients is reported to develop clinically apparent anterior scleritis at some point during follow-up [21, 24, 25]. The most commonly reported symptoms on presentation are periocular pain and headache (typically worse at night and waking the patient from sleep early in the morning), pain on ocular movement, and blurred vision [18, 21, 24, 25, 29]. It is important to note that painless presentations can occur. Systemic disease associations, including rheumatoid arthritis, systemic lupus erythematous and systemic vasculitis, are found in less than half of all patients [18, 21, 24, 25]. The most frequently reported clinical findings are conjunctival chemosis or hyperemia, anterior scleritis, anterior uveitis, choroidal folds, optic nerve swelling, serous retinal detachment and macular oedema [15, 18, 21, 24, 25]. Other presenting findings include a circumscribed subretinal mass in nodular posterior scleritis [13, 15, 16, 18, 24], annular choroidal detachment [15, 24], raised intraocular pressure including angle-closure glaucoma due to ciliochoroidal effusion [17, 30–34], retinal pigment epithelium (RPE)-rip [35], contiguous involvement of retinal artery and vein causing occlusion of the vessels [36–38], or stellate neuroretinitis [39]. As posterior scleritis can present with a wide range of symptoms and clinical findings, it can easily be overlooked or confused with other disease entities such as Vogt-Koyanagi-Harada disease (VKH), central serous chorioretinopathy (CSC) or choroidal malignancies. A high level of suspicion is thus warranted. Clinical characteristics and multimodal imaging findings of differential diagnoses of posterior scleritis are described in Table 2.

**Table 2:** Differential diagnosis of posterior scleritis

	Posterior scleritis	Central serous chorioretinopathy (CSC) [15, 40]	Vogt-Koyanagi-Harada disease (VKH) [15, 40, 41]	Choroidal melanoma^a^ [13, 40]
Typical clinical characteristics				
*Laterality*	Mostly unilateral	Unilateral or bilateral	Bilateral (second eye involvement within 2 weeks)	Unilateral
*Presenting symptoms*	Acute painful vision loss (typically worse at night or on eye movements)	Painless vision loss	Blurred vision, photophobia, ocular pain. Associated systemic symptoms depending on disease stage.	Painless visual changes (rarely painful when necrotic). Can be asymptomatic.
*Associated clinical signs*	Anterior scleritis, anterior uveitis, vitreous inflammation, choroidal folds, optic disc edema.	Round/oval serous retinal detachment with or without detachment of retinal pigment epithelium (RPE), RPE changes (focal or multifocal). No associated inflammatory signs.	Granulomatous anterior uveitis, vitritis, diffuse choroiditis, Dalen-Fuchs nodules, sunset glow fundus, optic disc edema. Frequent associated skin changes, CNS findings (incl. Cerebrospinal fluid pleocytosis)	Pigmented or amelanotic elevated choroidal mass, lipofuscin often present. Serous RD and/or sentinel vessel possible. Associated inflammation rare.
*Response to steroids*	Improvement of pain and imaging findings	Can worsen presenting signs and symptoms	Improvement of pain and imaging findings	None
Multimodal imaging findings				
*B-scan ultrasound*	T-sign, increased thickness of the posterior coats (> 2.0 mm), nodular subtype possible (sessile or dome shaped lesion with high internal reflectivity)	Serous RD possible	Serous RD possible	Dome-shaped or mushroom-shaped choroidal lesion with typically low to medium internal reflectivity. Choroidal excavation and serous RD possible.
*OCT*	Choroidal folds, serous retinal detachment, macular oedema	Subretinal fluid (SRF) (can be multifocal), RPE-detachment(s), intraretinal fluid (IRF) possible, atrophic RPE-changes possible	Serous retinal detachment with typical fibrinous septa	Dome-shaped solid choroidal mass, accompanying SRF possible
*EDI-OCT*	Localized choroidal thickening in affected eye in acute stages, choroidal thinning after treatment or in advanced stages	Diffuse choroidal thickening, dilated large vessels in Haller’s layer with thinning of overlying smaller vessels in Sattler’s layer and choriocapillaris	Choroidal thickening in acute stages, choroidal thinning after treatment or in advanced stages	Dome-shaped solid choroidal mass with smooth surface
*FA*	Early pinpoint leaks with late pooling in cases with subretinal fluid	Focal leak in acute phase (typically described as ‘ink blot’ or ‘smokestack’ leakage pattern). Multifocal leakage (‘hot spots’) and pooling under detached RPE possible. Disc leak absent.	Focal areas of delayed choroidal perfusion, multiple pinpoint regions of leakage at the RPE-level, disc hyperfluorescence	Multiple areas of pinpoint leakage
*ICGA*	Choroidal vasculitis, zonal choroidal hyperfluorescence (± pinpoint leakage), choroidal perfusion delay, enlargement of draining choroidal veins, and hypofluorescent dark dots	Early phase: large, dilated, densely packed choroidal vessels. Mid-to late-phase: choroidal vascular hyperpermeability (focal or multifocal hyperfluorescent staining with indistinct borders).	Early phase: hyperfluorescence. Early-to-mid-phase: hypofluorescence.	Mixed pattern of fluorescence, blockage of fluorescence in pigmented lesions

^a^Choroidal melanoma serves as an example of choroidal malignancies in this table. Other tumors such as metastatic deposits or choroidal lymphoma have different clinical and imaging characteristic and should also be borne in mind

The diagnosis of posterior scleritis can be aided by B-scan ultrasonography, which typically shows a T-sign due to the presence of fluid in the sub-Tenon’s space, along with increased thickness of the posterior coats (> 2.0 mm) [21, 24, 25, 29]. It is important to note that the absence of a T-sign does not exclude the diagnosis [24]. Magnetic resonance imaging (MRI) is known to have excellent soft tissue contrast and can be useful when the ultrasound findings are inconclusive. Scleral enhancement is the most commonly identified MRI-finding in posterior scleritis [42]. This can only be identified with the use of gadolinium contrast, thus the use of contrast is essential in suspected posterior scleritis. Scleral thickening and focal periscleral cellulitis are other important imaging findings on MRI [42–44]. CT imaging can also be useful in case of contraindications to MR imaging [42].

As posterior scleritis may cause pain and visual loss, and the risk of recurrences is high, early and aggressive treatment is recommended [11, 24]. Systemic non-steroidal anti-inflammatory drugs (NSAIDs) are the preferred first-line treatment in mild cases of anterior non-necrotizing scleritis, but in posterior involvement the use of systemic steroids (orally or intravenously) is usually recommended in view of the fact that it is not possible to predict the risk of visual loss even in cases that may appear to be mild in presentation [29, 45]. Immunosuppressive agents should be considered if the response to steroids is unsatisfactory, if long-term steroid sparing therapy is needed, or in case of recurrences [29, 45]. Methotrexate, Azathioprine and Mycophenolate Mofetil are most commonly used, while cyclosporin, cyclophosphamide, chlorambucil, and biologics such as anti-tumor necrosis factor alpha (anti-TNF-α) (e.g. Infliximab) or anti-CD-20 (e.g. Rituximab) can also be considered [45].

This review article aims to provide a comprehensive overview of choroidal involvement in non-infectious posterior scleritis, including different imaging modalities and their clinical usefulness in diagnosing and monitoring patients with posterior scleritis.

## Methods

A literature search of PubMed was performed using the search terms *‘posterior scleritis’, ‘scleritis’, ‘choroid’, ‘choroidal, ‘thickness’, ‘mass’, ‘vasculitis’, and ‘exudative retinal detachment’*. Only English articles were included, and no time restrictions were applied. Studies reporting solely infectious causes were excluded based on title and abstract, as infectious scleritis is outside the scope of this review. The references from the selected articles were scrutinized and additional relevant manuscripts were included.

## Results of literature search and discussion

Scleral inflammation can spread to the adjacent choroid and cause secondary choroidal involvement. Histopathological evidence on enucleated globes or biopsy specimens of posterior scleritis cases showed choroidal involvement in all seven cases reported by Calthorpe et al. [18] The choroid was reported to be thickened with inflammatory infiltrates. Choroidal vasculitis, onion skin thickening with occlusion of vessels, and adjacent focal RPE-lesions were also observed. Riono et al. reported multiple foci of non-necrotizing granulomatous inflammation of the choroid in one case of anterior scleritis with histologic features characteristic of sarcoid-related granulomatous inflammation [46]. A case report by Stacy et al. of a chorioretinal biopsy specimen in idiopathic progressing posterior necrotizing chorioretinitis confirmed diffuse granulomatous inflammation of the inner scleral layers with a small focus of necrosis, consistent with idiopathic autoimmune scleritis [47].

Further evidence of choroidal involvement can be observed by indocyanine green angiography (ICGA) changes in patients with posterior scleritis. Auer and Herbort reported the ICGA patterns that can be observed in patients with posterior scleritis, and identified areas of diffuse zonal choroidal ICG hyperfluorescence in the intermediate and late phases, presumably corresponding to the areas of maximal inflammatory activity [48]. These areas of ICG hyperfluorescence were reported to regress in response to treatment. ICGA can thus be a useful imaging modality to assess the extent of choroidal involvement, and to monitor disease progression and response to treatment.

For the purpose of this review choroidal involvement is divided into four categories based on clinical observations: (1) choroidal thickness, (2) choroidal vasculitis, (3) presentation as a choroidal or subretinal mass in nodular posterior scleritis, and (4) choroidal folds, choroidal effusion and exudative retinal detachment. These categories are not mutually exclusive and represent the spectrum of choroidal involvement in posterior scleritis.

### Choroidal thickness

**Fig. 1:**
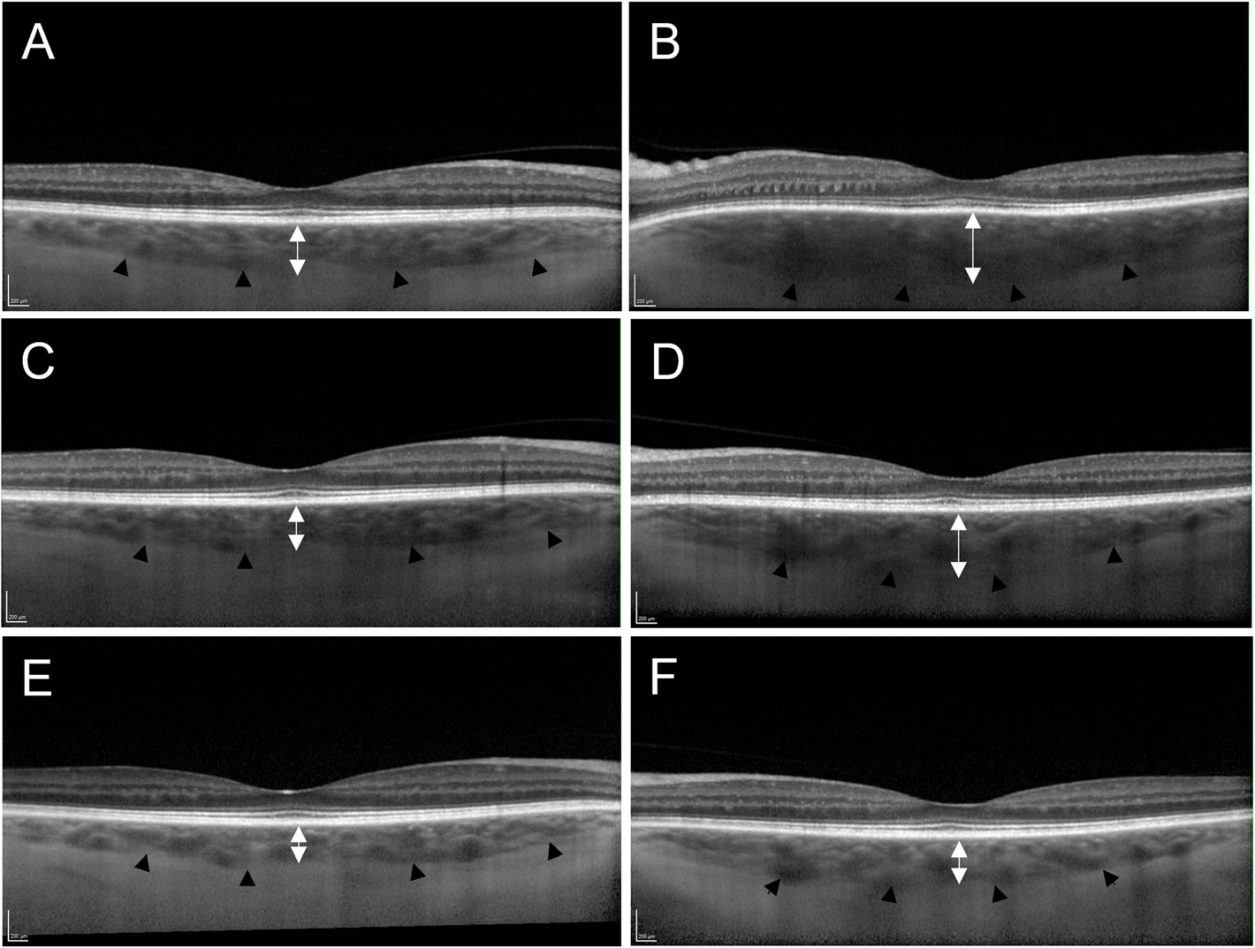
Enhanced-depth imaging optical coherence tomography (EDI-OCT) in a case of left posterior scleritis. The black arrow heads delineate the choroidal-scleral junction. (**B**) Spectralis EDI-OCT demonstrates left subfoveal choroidal thickening on presentation (442μm). The B-scan ultrasound showed thickening of posterior coats (2.5mm) and fluid in Tenon's capsule and optic nerve sheath (not shown). (**D**) Subfoveal choroidal thickness in the left eye decreased 1 week (406μm) and (**F**) 4 weeks (292μm) after initiation of oral prednisolone. (**A**,**C**,**E**) Normal subfoveal choroidal thickness in the clinically unaffected right eye of the same patient at corresponding points in time

While increased thickness of the posterior coats (> 2.0 mm) on B-scan ultrasound is a well-recognised diagnostic finding in posterior scleritis [21, 24, 25, 29], the involvement of the choroid in this process is less well studied. Recently, enhanced-depth imaging optical coherence tomography (EDI-OCT) has been used to characterise choroidal thickness in patients with posterior scleritis (Fig. 1). In a retrospective review published by Ando et al. subfoveal choroidal thickness, measured with EDI-OCT, was increased at presentation; and decreased with successful treatment during the follow-up period (mean choroidal thickness at presentation was 611 ± 148 μm, decreasing to 298 ± 75 μm (*p* < 0.01) at 1 month, and further decreasing afterwards) [14]. Dong and colleagues reported similar findings in a retrospective study of 23 patients with posterior scleritis and concurrent serous retinal detachment [20]. This case series showed an average subfoveal choroidal thickness with EDI-OCT of 442.61 ± 55.6 μm in the affected eye and 246 ± 42.3 μm in the unaffected eye (*p* < 0.01) and reported a significant correlation between subfoveal choroidal thickness and posterior coats thickness (as measured on B-scan ultrasound; r = 0.783, *p* < 0.001) and between subfoveal choroidal thickness and axial length (r = − 0.65, *p* = 0.001). Smaller case series confirm a marked subfoveal choroidal thickening in affected eyes with active posterior scleritis, with decreasing choroidal thickness after initiation of successful treatment, and increasing thickness if relapses occur [49, 50]. These results suggest that subfoveal choroidal thickness, measured with EDI-OCT, can be used as a biomarker for disease activity. This can be useful in diagnosing posterior scleritis and in monitoring the response to treatment. In eyes with frequent recurrences of posterior scleritis, choroidal thinning has been observed compared to the non-affected eye [50, 51]. This is presumed to be due to gradual choroidal atrophy due to severe or recurrent inflammation of the posterior sclera [50, 51].

### Choroidal vasculitis

Histopathological examination of scleral biopsies of patients with posterior scleritis can show active scleral vasculitis, as well as choroidal vasculitis, choroidal vascular closure and retinal vascular cuffing [18].

Adjacent choroidal changes in posterior scleritis can be evident on ICGA. Diffuse zonal choroidal ICG hyperfluorescence in intermediate and late phases was seen in all cases reported by Auer and Herbort, and regressed after anti-inflammatory treatment [48]. It could thus be hypothesized that the observed ICG hyperfluorescence is a representation of choroidal vasculitis adjacent to areas with active scleral inflammation. Other patterns that can be observed on ICGA in posterior scleritis are fluorescing pinpoints in the zonal hyperfluorescent areas, choroidal perfusion delay, enlargement of draining choroidal veins, and hypofluorescent dark dots up to the intermediate phase of the angiogram [48].

Choroidal vasculitis has been reported in a case report of nodular posterior scleritis associated with polyarteritis nodosa, and was evident on ICGA (segmental choroidal vessel staining and late leakage without choroidal ischemia) and OCT (hyperreflective thickening of the inflamed choroidal vessel wall) [52]. The authors reported disappearance of the choroidal vessel wall hyperreflectivity on OCT after anti-inflammatory treatment.

### Presentation as a choroidal or subretinal mass

**Fig. 2:**
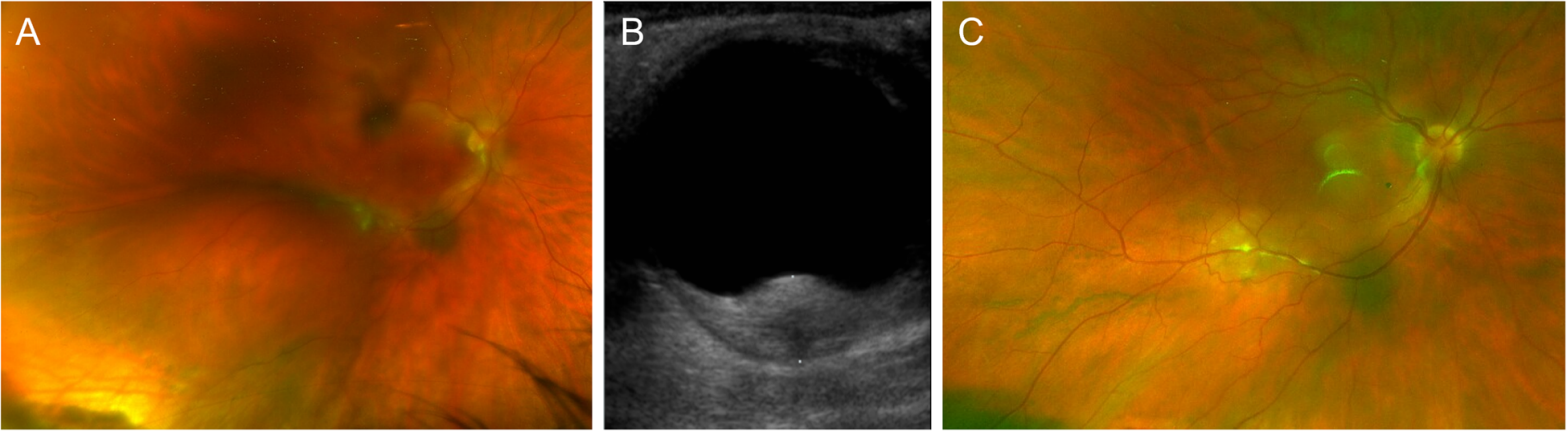
Selected imaging findings in nodular posterior scleritis. (**A**) Pseudocolour fundus photograph of nodular posterior scleritis. (**B**) Corresponding B-scan ultrasound findings, showing gross thickening of the posterior coats (6.6mm) with increased echogenicity. An ab externo biopsy was negative for malignancy. (**C**) The nodular lesion resolved with topical and systemic steroids, and Rituximab-infusions

Nodular posterior scleritis can present clinically as a choroidal or subretinal mass (Fig. 2), and can be difficult to differentiate from malignant choroidal tumors [13, 16]. Clinical characteristics can help differentiation: nodular posterior scleritis tends to be unilateral, can be associated with an underlying systemic disease, and is associated with intraocular inflammation in the majority of cases [13]. Moreover, nodular posterior scleritis is often associated with choroidal folds or macular edema, and can be seen as a solitary amelanotic mass without the presence of lipofuscin or drusen [13]. An overlying bullous serous retinal detachment with shifting fluid can be present [15]. B-scan ultrasonography is the key imaging investigation: nodular posterior scleritis presents as a solitary sessile or dome-shaped lesion with high internal reflectivity, associated subretinal fluid or a T-sign and no detectable blood flow [13].

Although pain is an important diagnostic element, it should be kept in mind, as mentioned above, that pain can be absent in posterior scleritis. It is therefore not a good differentiator between posterior scleritis and malignant choroidal tumors. If the diagnosis remains uncertain despite extensive noninvasive testing a choroidal biopsy may be required.

### Choroidal folds, choroidal effusion and exudative retinal detachment

**Fig. 3:**
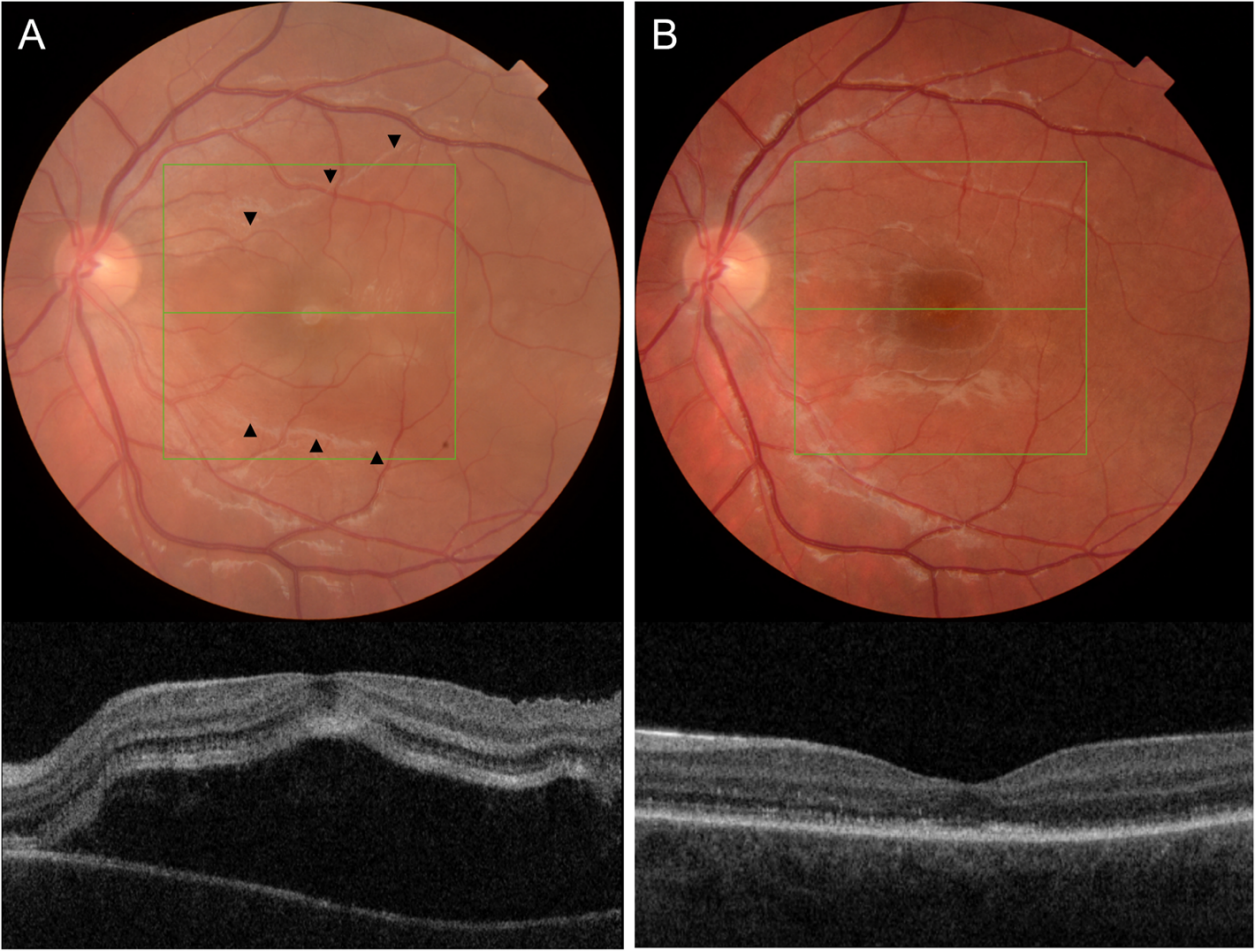
Serous macular detachment secondary to posterior scleritis. (**A**) Colour fundus photograph and Topcon optical coherence tomography (OCT) at presentation. Delineation of the exudative detachment can be seen on the colour fundus photograph (black arrowheads). (**B**) Resolution of the serous macular detachment 3 weeks after initiation of oral prednisolone

An exudative retinal detachment (ERD), or serous retinal detachment (Fig. 3), develops when fluid collects in the subretinal space due to disruption of the blood-retinal barrier [53]. This can be a complication of different types of ocular inflammation. In a retrospective review of uveitis-related ERD cases, Kinast and colleagues found that a serous retinal detachment was reported most frequently in Vogt-Koyanagi-Harada (VKH) disease (48.7%), while posterior scleritis was observed in 2.6% of all uveitis-related ERD-cases [54]. A similar result was found by Shah et al., who found 176 of 14,612 eyes (1.2%) with ocular inflammation presented with ERD [55]. The most frequent type of inflammation associated with ERD in this cohort was VKH as well (39.2%), while posterior scleritis was observed in 4.0%. Differentiating between VKH and posterior scleritis can be difficult, and a few case reports suggest the concurrent presence of both entities [41, 56, 57].

Inflammation of the sclera can lead to reduced permeability of the transscleral outflow, resulting in fluid accumulation in the choroid, while thickening of the sclera can contribute to compression of the vortex veins and thus cause hyperpermeability and congestion of choroidal veins, eventually creating a choroidal detachment [22].

The presence of choroidal folds, choroidal effusion and serous retinal detachment in patients with posterior scleritis varies between the retrospective cohort studies. Lavric and colleagues report choroidal folds and serous retinal detachments in 28.1% each [24]. Serous retinal detachments are, however, less frequently observed in posterior scleritis cases as reported by McCluskey et al. (21.0%), with choroidal effusion reported in 4.0% [25]. A higher prevalence of serous retinal detachments (39.1%) is observed by Calthorpe et al., with a ring choroidal detachment in 14.1% and choroidal folds reported in 10.9% in this retrospective study [18]. Benson and colleagues divided the clinical signs in their case series of posterior scleritis into different categories: 6 patients were observed to have choroidal folds, 7 patients had retinal striae and 1 had disc edema; furthermore an annular choroidal detachment was found in 7 patients, an exudative macular detachment in 15 patients, and a peripheral retinal detachment in one patient [15]. Dong et al. analyzed the clinical features of patients with a serous retinal detachment due to posterior scleritis [20]. In this retrospective study, a relatively young age at disease onset was reported (mean age 29.5 years old). Anterior scleritis was present in 52.0% of cases, which seems to be higher than in reports of posterior scleritis without serous retinal detachment [20]. Interestingly, Dong and colleagues reported that 74% of the patients in their study were initially misdiagnosed, underlining the importance of taking into account the clinical and imaging characteristics in such cases.

## Conclusion

A spectrum of choroidal changes in posterior scleritis can be observed. Active posterior scleritis can be associated with increased subfoveal choroidal thickness, which reduces again in response to anti-inflammatory treatment. Subfoveal choroidal thickness can thus be used as a biomarker for disease activity. The current evidence to support this is limited, and further studies are warranted to determine the prevalence of these findings and to corroborate the use of EDI-OCT in diagnosing and monitoring disease activity. Choroidal vasculitis can be seen on ICGA and possibly also on OCT. No studies were identified using OCT-angiography (OCTA) to characterize posterior scleritis, while this could potentially prove useful as suggested by the finding of a different degree of vascularity and tissue thickness with anterior-segment OCTA in anterior scleritis [58]. A high level of suspicion is warranted in cases of nodular scleral inflammation leading to the presentation of a choroidal or subretinal mass, as these can be difficult to discern from a malignant choroidal tumor. Clinical and imaging characteristics can be useful in differentiating a rather benign, inflammatory cause from a malignant process. Further studies are needed to better identify the role of new imaging modalities such as EDI-OCT and OCTA in diagnosing and monitoring posterior scleritis.

## Data Availability

Not applicable.

## Abbreviations

Anti-TNF-α: Anti-tumor necrosis factor alpha
CSC: Central serous chorioretinopathy
EDI-OCT: Enhanced-depth imaging optical coherence tomography
ERD: Exudative retinal detachment
FA: Fluoresceine angiography
ICGA: Indocyanine green angiography
MR: Magnetic Resonance
MRI: Magnetic Resonance Imaging
NSAIDs: Non-steroidal anti-inflammatory drugs
OCT: Optical coherence tomography
OCTA: OCT-angiography
RPE: Retinal pigment epithelium
SRF: Subretinal fluid
VKH: Vogt-Koyanagi-Harada disease
